# Quantification of the boron speciation in alkali borosilicate glasses by electron energy loss spectroscopy

**DOI:** 10.1038/srep17526

**Published:** 2015-12-08

**Authors:** Shaodong Cheng, Guang Yang, Yanqi Zhao, MingYing Peng, Jørgen Skibsted, Yuanzheng Yue

**Affiliations:** 1 Electronic Materials Research Laboratory, Key Laboratory of the Ministry of Education & International Center for Dielectric Research, Xi’an Jiaotong University, Xi’an, 710049, China; 2 State Key Laboratory of Luminescent Materials and Devices, South China University of Technology, Guangzhou, 510640, China; 3 Department of Chemistry and Interdisciplinary Nanoscience Center (iNANO), Aarhus University, Aarhus, DK-8000, Denmark; 4 Section of Chemistry, Aalborg University, Aalborg, DK-9000, Denmark; 5 State Key Laboratory of Silicate Materials for Architectures, Wuhan University of Technology, Wuhan, 430070, China

## Abstract

Transmission electron microscopy and related analytical techniques have been widely used to study the microstructure of different materials. However, few research works have been performed in the field of glasses, possibly due to the electron-beam irradiation damage. In this paper, we have developed a method based on electron energy loss spectroscopy (EELS) data acquisition and analyses, which enables determination of the boron speciation in a series of ternary alkali borosilicate glasses with constant molar ratios. A script for the fast acquisition of EELS has been designed, from which the fraction of BO_4_ tetrahedra can be obtained by fitting the experimental data with linear combinations of the reference spectra. The BO_4_ fractions (N_4_) obtained by EELS are consistent with those from ^11^B MAS NMR spectra, suggesting that EELS can be an alternative and convenient way to determine the N_4_ fraction in glasses. In addition, the boron speciation of a CeO_2_ doped potassium borosilicate glass has been analyzed by using the time-resolved EELS spectra. The results clearly demonstrate that the BO_4_ to BO_3_ transformation induced by the electron beam irradiation can be efficiently suppressed by doping CeO_2_ to the borosilicate glasses.

Borosilicate glasses have been widely used, for example, as thermal shock-resistant containers (e.g., Pyrex) and as substrate for liquid crystal displays (e.g., EAGLE XG)^1^,^2^,^3^. By changing the composition of borosilicate glasses the physical and chemical properties of these glasses can be tuned for targeted functionalities^4^. Alkali borosilicate glasses have also been used to immobilize high-level nuclear waste materials, including plutonium and uranium^5^. The radioactive elements can be imbedded in the glass matrix and thereby their exposure to the environment can be minimized. In borosilicate glasses, boron has two structural configurations, trigonal BO_3_ and tetrahedral BO_4_ units. The ratio of BO_4_ to BO_3_ has strong impact on the glass properties^4^ and several techniques have been developed to quantify the fraction of BO_4_ in borosilicate glasses, including X-ray absorption near edge structure (XANES)^6^,^7^, Raman spectroscopy^8^, nuclear magnetic resonance (NMR)^9^ and electron energy loss spectroscopy (EELS)^10^. Among these tools, ^11^B magic-angle spinning (MAS) NMR is one of the most reliable methods to quantify the molar ratio, N_4_ = [BO_4_]/([BO_4_] + [BO_3_]), in boron glasses. However, solid-state NMR instrumentation is not commonly found in the research laboratories. EELS can also be applied to quantify N_4_ since the BO_3_ and BO_4_ units exhibit characteristic features in the corresponding spectrum. However, the electron-beam irradiation effect is almost inevitable in the transmission electron microscope (TEM) and the N_4_ data have been reported to be lower than those measured by NMR, due to the transformation of BO_4_ into BO_3_ units during the signal acquisition^11^.

TEM has been applied to investigate the microstructure of various materials, such as ceramics, nano-particles, metals and thin films. However, TEM is not a popular technique to study glasses mainly due to the electron-irradiation damage. As insulators most glasses are not conductive in scanning electron microscope (SEM) and/or TEM instruments and thus, severe charging or heating effects can occur during the experiments, which may cause errors in the microstructural characterization. Previous studies have shown that the electron-beam irradiation damage could result in oxygen bubble formation, crystallization or phase separation in glasses^12^,^13^,^14^. In alkali borosilicate (ABS) glasses, one of the irradiation effects is the transformation of tetrahedral BO_4_ into planar BO_3_ units as a result of the removal of alkali species out of the illuminated area, and the speed of this transformation decreases with the size of the alkali ions increase (Li^+^, Na^+^, K^+^ and Cs^+^)^11^. It is reported that the experimental boron K-edge EELS spectra can be fitted using a combination of BO_3_ and BO_4_ reference spectra^11^. However, the obtained N_4_ values in this manner are always lower than those from the NMR measurements possibly due to the lack of fast EELS spectra acquisition and an insufficient fitting method. In this work, we have designed a script for DigitalMicrograph^15^ (software for the EELS data acquisition) to record the EELS spectra of the glass fragments upon beam illumination. We have also developed a quantification method for determining the N_4_ from the boron K-edge EELS spectra. To investigate the effect of chemical doping on the glass structure, we have prepared a CeO_2_ doped potassium borosilicate glass and analyzed its structural features.

## Results and Discussion

### Alkali borosilicate glasses

**Figure 1:**
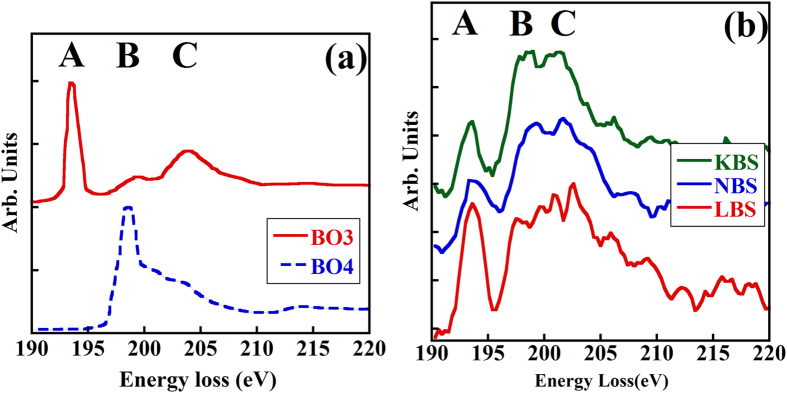
(**a**) Plots of boron K-edge reference fingerprint spectra of the BO_3_ and BO_4_ units found in the minerals vonsenite ([(Fe_1-x_,Mg_x_)O]_2_FeBO_3_) and rhodizite (M_0.9_Al_4_Be_4.55_B_11.35_O_28_), respectively^16^. (**b**) Experimental boron K-edge spectra of the LBS (red line), NBS (blue line) and KBS (green line) glasses.

The boron K-edge EELS spectra have fingerprints for different boron coordination environments, i.e., BO_3_ and BO_4_ units (see SI Fig. 1). Figure 1(a) illustrates the boron K-edge spectra (all spectra in this paper were background-subtracted and deconvoluted using the method details in the “Methods” part) of two reference materials that contain trigonal and tetrahedral boron, respectively^16^,^17^. There is one sharp peak at 193.6 eV and one broad weak peak at 204.1 eV in the BO_3_ spectrum, whereas one sharp peak at 198.6 eV in the BO_4_ spectrum. The intensity of these peaks defines the BO_3_ and BO_4_ fingerprints. According to molecular orbital (MO) theory^16^, the intense characteristic BO_3_ peak at 193.6 eV (peak A) is a π* peak, whereas the intense σ* peak at 198.6 eV (peak B) is the typical feature of BO_4_ units. The third peak at 204.1 eV (peak C) is attributed to both BO_3_ and BO_4_ configurations and this peak originates from the transition from the boron 1*s* state to the split σ* states for both boron coordinations^16^. It should be noted that the energy resolution of both reference spectra were about 0.5 eV, better than that of our experimental spectra (1.2 eV), which means that the peak height/width of our experimental spectra (Fig. 1(b)) may not be directly compared with the superposition of the two fingerprint spectra. In addition, the reference spectra were obtained for BO_3_ and BO_4_ units in minerals, where the bond lengths within the structural units may be different from those in the corresponding glasses^18^.Therefore, the sharp peaks in the reference spectra, which are sensitive to the energy resolution of the instrument and/or to the B-O bond lengths, may not be directly applied for N_4_ quantification. In the boron K-edge reference spectra, peak B is sharp in the BO_4_ reference spectrum and is sensitive to the B-O bond variations^16^, thus may not be suitable for the quantification of N_4_ in glasses. On the other hand, peaks A and C (Fig. 1) are observed in most boron containing materials and their shapes as well as intensities are relatively insensitive to the energy resolution of the microscope and to the B-O bond lengths. In this case peaks A and C are selected for the N_4_ quantification in the ABS glasses. Normally for the data quantification using EELS, the spectra from both reference materials and the studied materials need to be achieved from the same instrument. However, it is difficult to find either a glassy material with only single boron coordination (e.g. BO_4_) or the minerals used in the previous report^16^. We attempt to develop a method for quantifying the boron speciation using current available data and independent on the resolution of the TEM instruments. The reference boron K-edge EELS spectra in the previous report have been replotted and used as the references in this paper. Experimentally, the boron K-edge EELS spectrum in glasses exhibits a sharp peak at ~194 eV followed by a broad peak (Fig. 1(b)) covering both the B and C peaks (as indicated in the figure). Previous reports have shown that the BO_4_ units in glasses can be transformed into BO_3_ under the electron-beam irradiation^11^,^19^. For light alkali elements, such as Li, it is important to record the boron K-edge EELS spectrum at the beginning of the electron-material interaction since the stability of the alkali cations under the electron beam decreases as the atomic number decreases^11^. Figure 1(b) shows the experimental boron K-edge spectra of the lithium borosilicate (LBS) glass, sodium borosilicate (NBS) glass and potassium borosilicate (KBS) glass. All spectra are the first recorded ones acquired with fast electron-material interaction (~0.05 s), and they are normalized by peak B.

**Figure 2:**
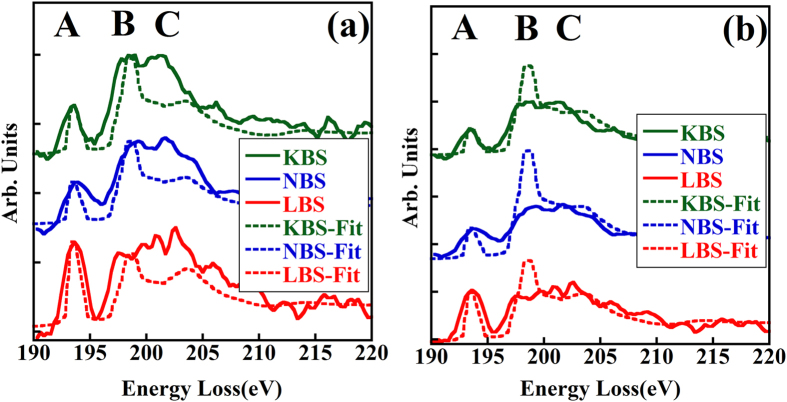
Experimental boron K-edge EELS spectra of the LBS, NBS and KBS glasses (solid lines) along with the fitted spectra (dashed lines) by (**a**) the previously reported method^11^ and (**b**) the fitting method developed in this paper.

From Fig. 1(b) it is clear that the LBS glass has a larger fraction of BO_3_ units than the NBS and KBS glasses (due to the higher peak A in LBS than that in NBS and KBS), and the similarity of the NBS and KBS spectra indicates that these glasses should have similar N_4_. First, we tried to utilize the reported method to quantify the N_4_ for the glasses^11^. As peak A is attributed to BO_3_ and peak B mainly originates from BO_4_ units, the N_4_ could be deduced from the intensity ratio of peak A and B by this method. Figure 2(a) shows the experimental results and the corresponding fitted spectra using the reference method. From the spectra, it can be seen that if the fitted spectra are modeled according to peak A and B, the resultant C peak seems to deviate from the experimental spectra. The N_4_ from the fitting are 49.1%, 63.9%, 62.1% for the LBS, NBS and KBS glasses, respectively. As mentioned in the experimental part, the acquired spectra were treated as the undamaged original structure and therefore in this case no time series extrapolation is needed for the N_4_ quantification (the time series was used in the previous report^11^, where the spectra were recorded and listed according to the irradiation time).

In this study we develop a method for quantification of the N_4_ ratio, where the intensities of peak A and peak C are used as references for the data fitting. This is motivated by the fact that the sharp peak A is dominated by the contribution from BO_3_ units and not from BO_4_ sites, while the peak C originates from both units and the different N_4_ would result in different ratios of peak A/C in the boron K-edge spectra. Therefore, it is worth fitting the experimental spectra with both the peak A and peak C intensities as shown in Fig. 2(b). The N_4_ obtained from the present method are 56.8%, 74.0% and 73.3% for the LBS, NBS and KBS glasses, respectively.

**Figure 3:**
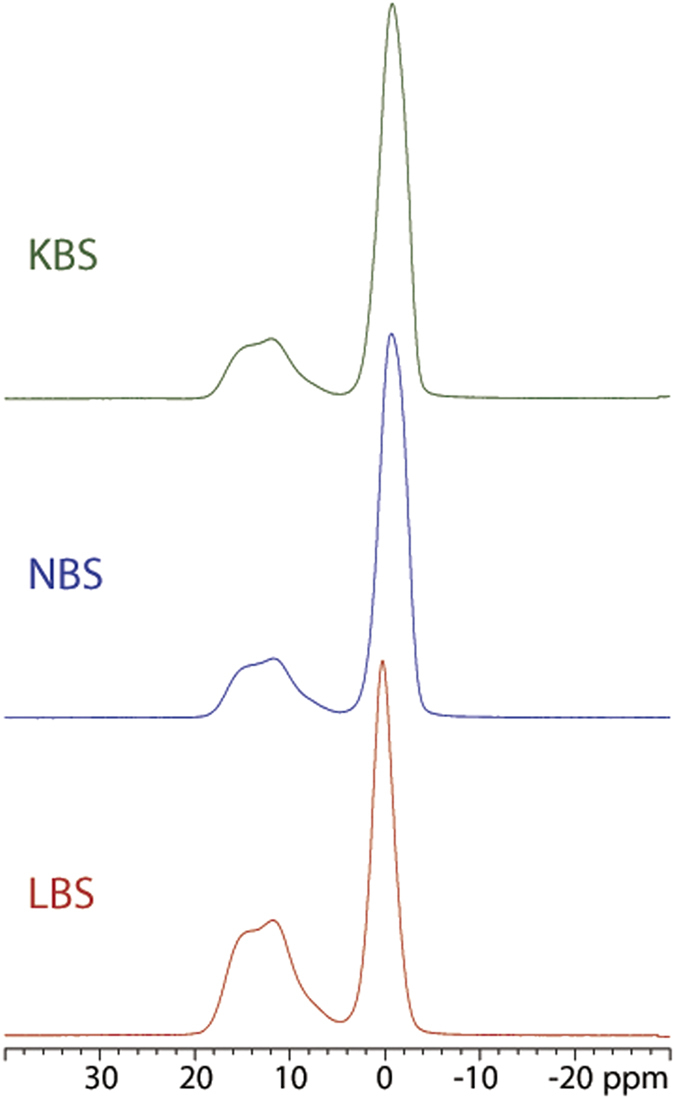
^11^B MAS NMR spectra of the LBS, NBS and KBS glasses, acquired at 14.09 T using a spinning speed of ν_R_ = 12.0 kHz.

^11^B MAS NMR spectra for the three glasses, illustrating the central-transition region, are shown in Fig. 3. The partly resolved second-order quadrupolar lineshape in the range 6–20 ppm originates from BO_3_ units, whereas the narrow peak at 0 ppm reflects BO_4_ sites. Spectra integration over the two resonances allows quantification of the fraction of BO_3_ and BO_4_ sites. For the LBS, NBS and KBS samples, this procedure gives N_4_ of 54.8%, 74.9% and 75.5%, respectively. All the N_4_ determined by the reported EELS method listed above, are much lower than the N_4_ from ^11^B MAS NMR, indicating that the previous reported EELS quantification method may not as good as the method developed in this study for the single spectrum N_4_ quantification.

The calculated N_4_ using the new quantification method (56.8%, 74.0% and 73.3% for the LBS, NBS and KBS glasses, respectively) are in an excellent agreement with those determined by ^11^B MAS NMR (54.8%, 74.9% and 75.5%, respectively). The small differences are within the range of the measurement errors for both the boron K-edge fittings and the ^11^B MAS NMR quantification, where the latter is estimated to be ±1.5% for the N_4_ of the studied samples. This indicates that peak C is more sensitive to the boron coordination rather than peak B. Other quantification methods, such as fitting peak B and C, or fitting of the experimental spectra using integrated intensities of peaks from pre-defined windows, were also tested but the resulting N_4_ had larger deviations than this data fitting method. The self-designed DM script combined with the curve-fitting method enables us to acquire boron K-edge spectra with minimum electron-beam damage, providing the basis for an improved determination of N_4_ by EELS.

### CeO_2_ doped potassium borosilicate glasses

**Figure 4:**
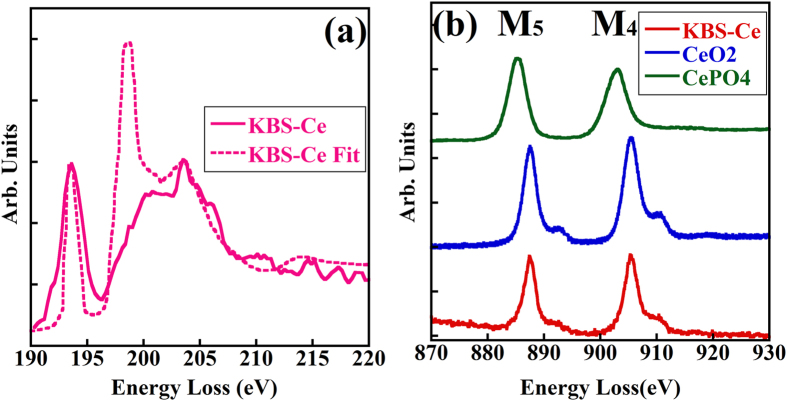
(**a**) Boron K-edge EELS spectrum (solid line) of the KBS-Ce glass and the fitted spectrum (dashed line); (**b**) Ce M_4,5_ edge EELS spectrum of the KBS-Ce glass and two reference spectra of CeO_2_ and CePO_4_.

In order to investigate the effect of oxide doping on the glass microstructure, we added 5 mol% CeO_2_ to the KBS glass (KBS-Ce). Figure 4(a) shows the boron K-edge EELS spectrum of the KBS-Ce glass, obtained under the same condition as those in Figs 1 and 2, along with the corresponding fitted spectrum. The latter gives the N_4_ of 60%, which is much lower than that for the KBS glass without CeO_2_ doping. The reason for this difference is still unclear, but it might be due to the possible octahedrally coordinated Ce^4+^ in the glass network that promotes the formation of interconnected boroxol rings as reported previously^18^,^20^.

According to the previous studies^21^,^22^, in some borosilicate glasses Ce possesses different valence states in precipitates and glass matrices. For the present KBS-Ce glass, no precipitate is found by either SEM or TEM analyses and therefore, Ce is considered to be completed dissolved in the glass matrix. Figure 4(b) shows the EELS spectra of the Ce M_4,5_ edges acquired in the KBS-Ce glass and two reference materials (CePO_4_ and CeO_2_ were used as Ce^3+^ and Ce^4+^ reference materials for Ce oxidation-state quantification, respectively). The valence states of Ce were quantified by the second-derivative method^23^ to be +3.96. Even though the error in the measurement of the Ce valence is estimated to be about ±(0.05 ~ 0.1), the peak-shift towards lower energy of the M_4,5_ edges indicates that there should be some Ce^3+^ions accompanying the major fraction of Ce^4+^ in the glass. This result is consistent with a previous study which shows that Ce favors the valence state of +4 when Ce is totally dissolved in the ABS glass matrix^19^,^21^. The glass composition, the types and amounts of dopants, the melting condition as well as precipitates could affect the Ce valence states in the glass.

**Figure 5:**
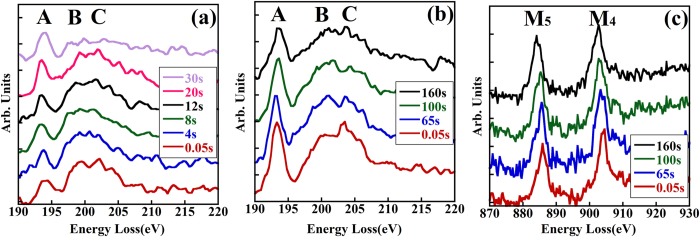
Electron-beam irradiation time series of (**a**) boron K-edge spectra from KBS glass and (**b**) boron K-edge spectra from CeO_2_ doped KBS glass; (**c**) Ce M_4,5_ edges in CeO_2_ doped KBS glass. The listed numbers indicate the exposure time of the glasses to the electron-beam.

Glasses are usually considered to be electron-beam sensitive. Borosilicate glasses are often doped with certain types of metal oxide for the sake of nuclear waste immobilization. However, it is not clear how the dopants affect the microstructure upon irradiation. The SiO_4_ tetrahedra in the alkali borosilicate glasses are stable under the electron beam (no observable fine structure variation within the experimental time), whereas the BO_4_ units are converted to BO_3_ units. Figure 5(a,b) show the electron-beam irradiation time series of boron K-edge spectra for the glasses with or without CeO_2_ doping. All spectra are normalized using peak A. The electron-irradiation durations for the glasses are indicated on the spectra. In the KBS glass time series (Fig. 5(a)), the relative intensity of the peak caused by BO_4_ (i.e., peak B) rapidly decreases with time, and after 30 s peak B almost disappears. However, in the CeO_2_ doped KBS glass peak B is still visible even after 160 s. This indicates that the addition of CeO_2_ enforces the electron irradiation resistance of the BO_4_ network. Figure 5(c) shows the Ce M_4,5_ edge spectra of the KBS-Ce glass for different irradiation durations. The signal-to-noise ratio (SNR) in the spectra is worse than that in Fig. 4(b), which may reflect the short acquisition time (~1 s for each spectrum and the first spectrum was acquired after 0.05 s electron exposure). However, it is clear that upon electron irradiation the M_4,5_ edges shift to lower energy, indicating the decrease in valence state of Ce in the glass. The Ce valence states are quantified to be +3.96 to +3.86 from the initial and final spectra, respectively. This means that the Ce ions act as a buffer to retard the damage of the glass network during electron-beam irradiation. The Ce^4+^ ions could absorb electrons which thereby are transformed into lower valence ions, and hence reducing the BO_4_ to BO_3_ transformation induced by the electron ionization damage.

In order to achieve a high enough SNR ratio of the boron and Ce time series EELS spectra, the electron probe was focused onto the glass particles during data acquisition. In other words, the electron dose was much higher in this study than in normal TEM image/spectra analysis. The focused electron probe enables collection of more signals by the spectrometer while accelerating the electron-irradiation damage process. This may explain why in some studies the valence states variation of the metal could not be directly observed by EELS at low or mediate electron dose rates.

In summary, we have designed a script in DigitalMicrograph and applied it to record boron K-edge EELS spectra of lithium-, sodium- and potassium-borosilicate glasses. The structure of these glasses is sensitive to electron-beam irradiation, and this is manifested by the transformation of tetrahedral BO_4_ units into planar BO_3_ units under the electron beam. The EELS spectra with short irradiation time (~0.05 s) were recorded and quantified by fitting the peaks at 193 eV and 204 eV to a linear combination of two reference spectra and the BO_4_ factions were found to be consistent with those determined by ^11^B MAS NMR. To investigate the effect of metal oxides on the microstructural stability of glass under the electron beam irradiation, 5 mol% CeO_2_ was added to the potassium borosilicate glasses. The BO_4_ fraction in the KBS-Ce glass was found to be lower than that in the KBS glass and the Ce valence state was quantified to be close to +4 by the second-derivative method. The EELS spectra for the electron-beam irradiation time series reveal that after CeO_2_ addition, the glass microstructure becomes more resistant to the electron-beam irradiation possibly due to a transition of Ce^4+^ to lower valence state. In short, we have developed a reliable method based on electron energy loss spectroscopy to quantify the boron speciation in borosilicate glasses, and the measured N_4_ are consistent with those obtained from ^11^B MAS NMR data. This work has presented an alternative approach for quantifying the boron speciation in glass materials.

## Methods

### Materials synthesis

Previous research has shown that the caesium borosilicate glasses are stable under the electron-beam irradiation^11^. Therefore the glass composition in this study was chosen as A_2_O:B_2_O_3_:SiO_2_ = 20:20:60 (mol%), where A = Li, Na and K. The lithium-, sodium- and potassium-borosilicate glasses are denoted as LBS, NBS, KBS, respectively (glass composition is summarized in Table S1 in the Supporting Information). For this composition, *R* = [A_2_O]/ [B_2_O_3_] = 1 and *X* = [SiO_2_]/ [B_2_O_3_] = 3. For all glass melts, reagent-grade alkali carbonates, boric acid and silica sand were used to give 200 g glass melt. 3 mol% excess H_3_BO_3_ was intentionally included to compensate for the boron loss due to volatilization of boron at high temperatures. The melt was homogenized at 1200 °C for 2 hours with 1 hour stirring. Afterwards, the glass melts were poured into a preheated steel mould and transferred to a furnace where the formed glasses were annealed at 570 °C for 1 hour and then cooled to room temperature. All glasses are confirmed to be amorphous by X-ray diffraction (XRD) analysis, and the LBS glass optically exhibits an opal color rather than being transparent. The cerium-doped glass was made by adding 5 mol% CeO_2_ to the potassium borosilicate glass mix (KBS-Ce, *R* = 1, *K* = 3) and synthesized by the same procedure as described above. The KBS-Ce glass is optically transparent and no precipitates were found by SEM and TEM analyses.

### Microstructure characterization

To minimize the possible ion-beam damage of glasses during sample preparation, the TEM specimens used in this study were prepared by first crushing the bulk glasses using an agate mortar and pestle with methanol, and then placing a drop of liquid containing glass fragments on a lacey carbon film covered copper grids. The electron energy loss spectroscopy experiments were conducted on a Gatan Enfina spectrometer mounted on an aberration corrected JEOL JEM ARM200F transmission electron microscope operated at 200 kV. Routinely, 1.2 eV at the dispersion of 0.3 eV/pixel can be achieved of the full width at half maximum (FWHM) of the zero loss peak (ZLP).

In this study, a plug-in script for the DigitalMicrograph^15^ software was designed specifically for the acquisition of EELS spectra for beam sensitive samples^24^. The script works in the following way. In TEM mode, the specimen is moved randomly while the EELS spectra are acquired with short exposure time (~0.05 s). The script can save all spectra automatically in a predefined folder. When the glass fragment is accidentally moved into the observation area, if the fragment is thin enough, a boron K-edge peak appears in the EELS spectrum. Once the boron edge appears, the movement of the specimen is immediately stopped and the acquisition continues until manual termination. For each sample several time series spectra have been recorded, and by eliminating those obtained from thick regions, the remaining spectra were background-subtracted by fitting the pre-edge to a power law function *AE*^–*r*^, where *E* is the energy loss and *A* and *r* are constants^25^. All the spectra were deconvoluted by their corresponding ZLP and low-loss spectra using the Fourier ratio method^26^. The energy scale of all spectra was calibrated according to the energy threshold of the C K-edge at 284 eV.

The ^11^B MAS NMR spectra were acquired at 14.09 T on a Varian Direct-Drive VNMRS-600 spectrometer, using a homebuilt CP/MAS probe for 4 mm o.d. zirconia (PSZ) rotors. The experiments employed a spinning speed of 12.0 kHz, a 0.5 μs excitation pulse for a radio-frequency (rf) field strength of γ*B*_1_/2π = 60 kHz, and a 4 s relaxation delay. The ^11^B isotropic chemical shifts are in ppm relative to neat F_3_BO(CH_2_CH_3_)_2_.

## Additional Information

**How to cite this article**: Cheng, S. *et al.* Quantification of the boron speciation in alkali borosilicate glasses by electron energy loss spectroscopy. *Sci. Rep.* **5**, 17526; doi: 10.1038/srep17526 (2015).

## Supplementary Material

Supplementary Information
