# Supplementary material for: Quantification of the boron speciation in alkali borosilicate glasses by electron energy loss spectroscopy
Source: Sci Rep. 2015 Dec 8;5:17526. doi: 10.1038/srep17526 (PMC4672332; doi:10.1038/srep17526)
Supplement: Supplementary Information [file srep17526-s1.doc]

**Supporting Information**

**Quantification of the boron speciation in alkali borosilicate glasses by electron energy loss spectroscopy**

Shaodong Cheng^1^, Guang Yang^1^,*, Yanqi Zhao^2^, MingYing Peng^2^, Jørgen Skibsted^3^, Yuanzheng Yue^4,5^

^1^Electronic Materials Research Laboratory, Key Laboratory of the Ministry of Education & International Center for Dielectric Research, Xi’an Jiaotong University, Xi’an, 710049, China

^2^State Key Laboratory of Luminescent Materials and Devices, South China University of Technology, Guangzhou, 510640, China

^3^Department of Chemistry and Interdisciplinary Nanoscience Center (*i*NANO), Aarhus University, Aarhus, DK-8000, Denmark

^4^Section of Chemistry, Aalborg University, Aalborg, DK-9000, Denmark

^5^State Key Laboratory of Silicate Materials for Architectures, Wuhan University of Technology, Wuhan, 430070, China

[*g.yang@mail.xjtu.edu.cn](mailto:*g.yang@mail.xjtu.edu.cn)


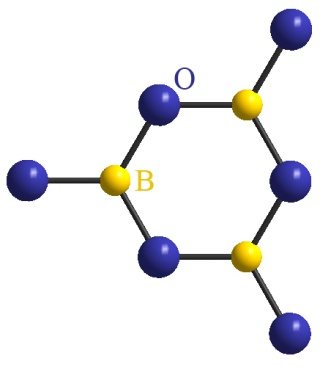

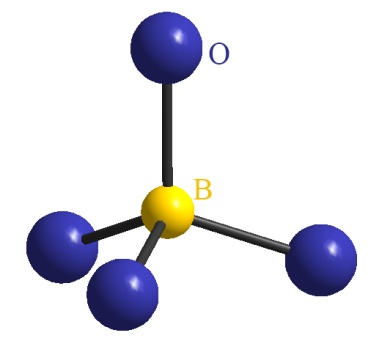


(a) (b)

**Figure S1** Schematic diagram of (a) planar BO_3_ and (b) tetrahedral BO_4_ unit

| Glass composition | A_2_O (mol%) | B_2_O_3_(mol%) | SiO_2_(mol%) | CeO_2_ (mol%) |
| --- | --- | --- | --- | --- |
| LBS | 20 | 20 | 60 |  |
| NBS | 20 | 20 | 60 |  |
| KBS | 20 | 20 | 60 |  |
| KBS-Ce | 19 | 19 | 57 | 5 |

**Table S1** The composition of glasses used in this paper. The LBS, NBS and KBS denotes the lithium-, sodium- and potassium-borosilicate glasses, respectively. The A_2_O indicates the alkali oxides.
